# Mental Toughness and Resilience in Trail Runner’s Performance

**DOI:** 10.1177/00315125231165819

**Published:** 2023-03-24

**Authors:** Nuno Gameiro, Filipe Rodrigues, Raúl Antunes, Rui Matos, Nuno Amaro, Miguel Jacinto, Diogo Monteiro

**Affiliations:** 1 70867 ESECS – Polytechnic of Leiria, Leiria, Portugal; 2 Life Quality Research Centre (CIEQV), Leiria, Portugal; 3 Center for Innovative Care and Health Technology (ciTechCare), 70866 Polytechnic of Leiria, Leiria 2411-901, Portugal; 4 Research Centre in Sports Sciences, Health Sciences and Human Development (CIDESD), Vila Real, Portugal

**Keywords:** endurance, mental toughness, performance, resilience, trail running

## Abstract

Our purpose with this study was to analyze trail runners’ psychological variables of mental toughness (MT) and resilience, and their associations with runners’ performances within a quantitative cross-sectional study. In total, we analyzed data from 307 Portuguese trail runners (60 female, 247 male), aged between 20 to 66 years (*M* age = 41.98; *SD* = 7.74). The results showed that the measurement model, including the factors of MT, resilience, and performance variables, exhibited an adequate fit to the data: *χ*^*2*^ = 150.01 (74); *BS-p* = .003; CFI= .953; TLI = .942; RMSEA = .058 90% (.045, .071) and SRMR= .042. Standardized direct effects revealed positive associations between these variables. More specifically: (a) MT was significantly associated with resilience; and (b) resilience was significantly associated with performance. The indirect regression paths showed that MT was positively associated with performance, with resilience considered a possible mediator (*β* = .09 IC = .010, .168; *p* = .02). In total, considering direct and indirect effects, the model explained 21% of performance variance among trail runners.

## Introduction

Exercise benefits have been well described in past literature (Spittler & Oberle, 2019) with agreement that exercise contributes favorably to the physiological, psychological, and social development of the human being (Bostancı et al., 2019). Traditional recreational running is a very popular form of exercise around the world (Waśkiewicz et al., 2019) creating positive lifestyles changes (Gajardo-Burgos et al., 2021). Over recent years, there has been increasing interest in running longer distances (Spittler & Oberle, 2019), probably due to modern societal demands to overcome adversities and challenges (Goddard et al., 2019). Parallel to this tendency, there is an increase in the athlete´s participation in off road or track endurance races known as trail running (Easthope et al., 2014; Malliaropoulos et al., 2015; Suter et al., 2020), involving unsurfaced mountain trails with extensive vertical displacement and different distances (Easthope et al., 2014; Malliaropoulos et al., 2015). Trail running is an outdoor sport (Viljoen et al., 2022) that is becoming one of the most popular disciplines in endurance running (Groslambert et al., 2020; Scheer et al., 2019). The International Association of Athletics Federations has recognized trail running as a new running discipline (Perrotin et al., 2021) and there are an estimated 20 million trail runners, with increased participation in the last decade (Viljoen et al., 2022). With a strong sense of sports ethics and a sense of humility, trail running is a sport that takes place amid nature and respects the environment as demanding for both body and mind (International Trail Running Association [ITRA], 2022). Trail running races are characterized by pedestrian competitions open to everyone, with a minimum use of paved roads (20% maximum) (Malliaropoulos et al., 2015) and distances that can range from a few kilometers to more than 200 km (in a multiday marathon) (Viljoen et al., 2022). These endurance races occur in variable terrains, including over frequent significant climbs and descents (elevation gain and loss), in varied environmental conditions like cold, heat, high altitude, snow, and humidity conditions (Perrotin et al., 2021) creating high overall difficulty for a given race (ITRA, 2022). The conditions can influence both the biomechanical and psychological state of the runner and, therefore, the overall performance during the race (Perrotin et al., 2021). Thus, there is a need to attend to the runner’s senses, their age, and the runner’s medical condition to explore their capabilities and develop their physical and mental abilities (ITRA, 2022). Recently, trail running has become more accessible to non-professional athletes, despite its demands and requirements for training, work schedule and personal life sacrifices (Rochat et al., 2017). In addition, several events or changing dynamics may occur during a race (Suter et al., 2020) due to the many variable exigencies of this sport (Scheer et al., 2019), including a combination of the runner’s own physical, tactical, and psychological characteristics that can lead to success or failure (Liew et al., 2019). In trail running, as in all other sports, performance is influenced by many physical characteristics like toughness, strength, speed, agility, and by the athletes’ psychological status such as motivation, concentration, and mental ability (Namli & Demir, 2019). In this regard, toughness may be needed for correct decision making under stress-difficult conditions, staying calm under pressure, and overcoming troubles that are intertwined with performance; this makes mental toughness a critical competency for achieving best success (Namli & Demir, 2019).

A characteristic of activity endurance is surely the demand for mental and physical reintegration following fatigue induced by the performance effort (Diotaiuti et al., 2021). Especially since performance can be challenged by unpredictable occurrences like weather conditions, mechanical failures, pain, or discomfort related to physical and mental states (Diotaiuti et al., 2021), it is imperative that athletes prepare in a comprehensive but focused way (Scheer et al., 2019). The influence of psychological factors in endurance sports is of undeniable importance; and, while there has been growing interest in studying these factors, and their impact on performance, they have been poorly analyzed to date (Méndez-Alonso et al., 2021), leaving us with insufficient data of this kind (Scheer et al., 2019). Different types of mental resources are apt to be important for an athlete’s preparations for the exigencies and challenges of this sport (Moreira et al., 2021).

### Mental Toughness

Mental toughness (MT) is a psychological construct with demonstrated importance in sport (Brace et al., 2020; Cooper et al., 2020; Jones & Parker, 2019) and there has been increased research and practice interest on mental toughness in sport and exercise psychology over the last two decades (Gucciardi, 2017). MT has been associated with beneficial behaviors and better sport performance outcomes (Stamatis et al., 2020), though there is still inadequate consensus regarding its conceptualization (Hardy et al., 2014). Amongst several definitions of MT, Gucciardi et al. (2015) defined it as “a personal capacity to produce consistently high levels of subjective (e.g., personal goals or strivings) or objective performance (e.g., sales, race time, GPA) despite everyday challenges and stressors as well as significant adversities.” Zeiger & Zeiger (2018) defined MT as “a state-like psychological resource that is purposeful, flexible and efficient in nature for the enactment and maintenance of goal-directed pursuits,” such that MT enables striving (e.g., effort), surviving (e.g., coping) and thriving (e.g., performing) (Jones & Parker, 2019). MT shares conceptual space and has been correlated with various other psychological constructs (Brace et al., 2020; Jones & Parker, 2019) like optimism, pessimism, coping, youth experiences, achievement goals and sport motivation, developmental assets, and stress appraisal (Jones & Parker, 2019), as well as resilience, self-belief, and emotional intelligence (Nicholls et al., 2015). Nicholls et al. (2015) suggested that it might be the presence of other psychological constructs (rather than coping alone) that permits mentally tough individuals to distinguish themselves under stressful circumstances (Nicholls et al., 2015). Evidence suggests that MT is a multifaceted construct that supports performance excellence (Cowden et al., 2017; Shaari et al., 2020) irrespective of the type, direction, and degree of demands experienced (Cowden et al., 2017). Additionally, MT is considered central to sport performance (Anthony et al., 2016) and it is an important prerequisite for a higher sustained athletic performance (Goddard et al., 2019). MT is classified as a critical factor for success (Souter et al., 2018) because of its role in increasing a controlled adaptative response to positive and negative pressures, situations, and events (Cowden et al., 2017). The implicit association between MT and success or better performance has gained increasing attention among leading researchers, especially those who have conducted retrospective studies of elite athletes (Cowden et al., 2017). Despite the promising potential of developing MT, there is no evidence to date for any specific approach to training this attribute. Nevertheless, Stamatis et al. (2020) suggested that due to sport-specific differences in MT, interventions to enhance MT should consider the cultural and contextual attributes of each sport. Additionally, some investigators have begun to quantify the predictive role of MT for competitive (Cowden, 2016) and noncompetitive (Gucciardi et al., 2016) performance indicators. These results have not been discussed in detail and there is still a scarcity of empirical research regarding this conceptual association between MT and athletic performance. More specifically, it is still unclear whether MT is noticeable in athletes with better performance, higher achievement, or successful outcomes, or whether MT is more apt to be evident in association with non-performance factors like resilience, self-belief, and emotional intelligence (Cowden et al., 2017). Cowden et al. (2017) highlighted the importance of conducting statistically based studies that more accurately control the relationship between levels of MT on performance outcomes. Stamatis et al. (2020) highlighted the importance of conducting studies with objective indicators of athletic performance to provide evidence for MT interventions.

### Resilience

As previously noted, another construct that is frequently mentioned alongside MT is resilience (Liew et al., 2019). Resilience in sport has aroused interest due to athletes’ needs to use and optimize a range of mental qualities to protect them from or to overcome stressors, adversities, and failures (Galli & Gonzalez, 2015; Sarkar & Fletcher, 2014). Sport is an excellent context in which to study resilience for coping with unexpected adversities like serious injuries, or stressors of a psychosocial nature (e.g., losing a match, maladaptive interactions with coach), a physiological nature (e.g., high training loads) or a non-typical circumstance (e.g., pandemic situations) (den Hartigh et al., 2022). Additionally, athletes submit themselves continuously to evaluative environments with high consequences (e.g., winning or losing) (Galli & Gonzalez, 2015). Thus, to distinguish psychological resilience from other forms of resilience, Fletcher and Sarkar (2012) first defined resilience as “the role of mental processes and behavior in promoting personal assets and protecting an individual from the potential negative effect of stressors.” Additionally, resilience has been seen as the individual’s capacity to recognize personal limits, and to accept and go further to face difficulties with optimism (Diotaiuti et al., 2021). Roebuck et al. (2020) conceptualized resilience as a “high-order trait that reflects the ability of a person to maintain normal psychological functioning in the setting of a stressor.” There still exists controversy between the definition and the concept of resilience in research and sport practice, with some investigators noting continued difficulties operationalizing and measuring resilience, both in this context and in non-sport settings (Galli & Gonzalez, 2015). Resilience has been studied, with multidisciplinary interest, as a dynamic process with a personalized perspective (den Hartigh et al., 2022), leading sport scientists to adopt one of two possible approaches (Galli & Gonzalez, 2015). On one hand, they have examined the psychosocial factors that predict performance following an initial failure on the same task, seeing resilience as a coping behavior characterized by performing successfully after an initial failure or trying after-the-fact to identify how to enhance resilience/performance (Galli & Gonzalez, 2015). From the other perspective, resilience has been investigated by attempting to understand the thoughts, beliefs, emotions, and behaviors of athletes who demonstrate a capacity to overcome adversity in sport (Galli & Gonzalez, 2015). Nonetheless, the athlete’s personal qualities of resilience (Galli & Gonzalez, 2015) namely positivity, determination, competitiveness, commitment, maturity, persistence, passion for the sport (Sarkar & Fletcher, 2014) as well as social and environmental contexts appear to have an important role (Galli & Gonzalez, 2015). In qualitative studies, researchers have focused on psychological elements that protect athletes against stressors; they have emphasized positive personality, motivation, confidence, focus and perceived social support as main protective factors (Sarkar & Fletcher, 2014). Additionally, positive personality traits, and, more specifically, adaptative perfectionism, optimism and competitiveness have been linked with dealing with stressors (den Hartigh et al., 2022). Consequently, studying this dynamic process of bouncing back to normal functioning following adversity and noting specifically how long this takes have been seen as keys to understanding how to prevent performance depletion and contend with psychological or physical stresses (den Hartigh et al., 2022). Utilizing biopsychosocial data, it is possible to determine warning signs of a loss of resiliency (den Hartigh et al., 2022).

### Current Integrative Research

As noted above, resilience can interact with other psychological constructs in sport like hardiness, coping, MT, and post traumatic growth (Galli & Gonzalez, 2015). Thus, Gucciardi et al. (2008) and Nicholls et al. (2015) focused on the relationship between resilience and MT in sport and, more recently, Moreira et al. (2021) showed that, while MT incorporates characteristics of resilience and hardiness, it also enables one to thrive in situations where there are positive effects and perceived positive pressure. Resilience is very similar to MT (Cowden et al., 2016) and both constructs have very often been cited together (Liew et al., 2019; Gucciardi et al., 2009). Like MT, conceptualization, operationalization, and measurement of resilience have not yet generated a consensus (Cowden et al., 2016). While they share similar conceptual space, their relationships to each other have not been explicitly clarified (Nicholls et al., 2015). Nonetheless, it is important to clarify some dissimilarities. Clough et al. (2002) proposed that confidence, a component of MT, is the distinguishing factor between both constructs. Anthony et al. (2020) also underlined the importance of resilience. For instance, Anthony et al. (2020) showed that resilience could act as an emergent outcome, both in terms of individual and group systems, that allows one to bounce back quickly to homeostasis following adversity; whereas MT is only related to the psychological capacity of individuals or resources, acting as a protective factor. Aryanto and Larasati (2020) pointed to the fact that resilient individuals control their behavior by remaining focused, despite identifying and controlling negative influences, while MT individuals can reject outside negative effects to the point that they are unaware of them. MT could be applied to positive circumstances, representing a group of personal attributes that impact the way in which adversity, challenges and goals are surrounded and assessed (Cowden et al., 2016). On the other hand, resilience is mostly associated with negative contexts, including possession of and/or the presence of protective and vulnerability factors, such that resilience influences the relationship between risk and positive adaptation and may influence and be influenced by important attributes outside of the self (e.g., perceived social support) (Cowden et al., 2016). Nonetheless, Cowden et al. (2016) emphasized the studies conducted by Gerber et al. (2013) in which MT was seen as “a resilience resource or protective factor that moderated the association between risk and adaption levels to facilitate positive outcomes.”

In line with the inherent growth of interest in sports generally, the growing interest in trail running races by sport professionals has contributed to increased attention to the characteristics that athletes might develop through training and competitions to better their race performances (Méndez-Alonso et al., 2021). In this way, owing to the limitations of physical training, the possibilities and seeming limitlessness of psychological training has become a newer crucial focus (Zeiger & Zeiger, 2018). Given the promising results of MT (Méndez-Alonso et al., 2021; Guszkowska & Wojcik, 2021) and resilience (Diotaiuti et al., 2021; Galli & Gonzalez, 2015) constructs in endurance performance sports, endurance runners have begun to try to raise their levels of tenacity, determination, and tolerance of negative affect (e.g., resilience traits) (Diotaiuti et al., 2021). Nonetheless, quantitative studies analyzing these constructs through validated measurement instruments in endurance sports like trail running are still scarce.

In the present study, our objective was to analyze these psychological variables among trail runners and to study the association between these variables and athletic performance as measured by the International Trail Running Association Index (ITRA Index). More specifically, we studied the relationship between MT and resilience and athletic performance, hoping to contribute a quantitative study that would lend a better understanding of these two constructs, outlining their similarities and differences.

## Method

### Participants

A total of 307 Portuguese trail runners (60 female, 247 male), aged between 20 to 66 years (*M* age = 41.98; *SD* = 7.74) participated in this study Data were collected in accordance with the Helsinki Declaration World Medical Association (2013); and the Ethics Committee of the Polytechnic of Leiria gave its approval for its implementation (CE/IPLEIRIA/26/2021). Potential participants were contacted though the Portuguese Trail Running Association platform. Additionally, social network pages, forums, and individual teams were contacted. A Google form was sent to all potential participants of this study. To be included in this study, potential participants needed to obtain a valid ITRA profile. This means that athletes needed to have finished at least one certified ITRA race in the previous 36 months. Before data collection, potential participants were informed about the main objectives of the study, and the estimated time to complete the assessment battery (approximately 12 minutes). Before completing the questionnaires, participants had to complete a check box, ensuring that they understood the aims of the study, and that they consented to participate. Participants were thanked for their contribution, but no compensation was provided.

### Measures

#### Mental Toughness

In this study, we used the Sport Mental Toughness Questionnaire (SMTQ) developed by Sheard et al. (2009) in a Portuguese version by Fonseca (2012). The SMTQ was established to ascertain the athlete´s mental toughness levels. This questionnaire is comprised of 14-items that are answered on a four-point Likert-type scale, ranging from “not at all true” [1] to “very true” [4]. The items are grouped into three factors: Confidence – six items (e.g., “I interpret threats as positive opportunities.”); Constancy – four items (e.g., “I give up in difficult situations.”) and Control – four items (e.g., “I am overcome by self-doubt.”). Previous studies supported the validity and reliability of this measure among athletes (Sheard et al., 2009; Zeiger & Zeiger, 2018). For the present study, we utilized the three factor constructs (Confidence, Constancy, and Control) representing mental toughness (Miçooğulları, 2017).

#### Resilience

The 10 item Connor-Davidson Resilience Scale (CD-RISC-10) developed by Connor and Davidson (2003) is available in a Portuguese version adapted by Almeida et al. (2020). The CD-RISC-10 measures resilience in the general population, and it has been tested among athletes with adequate psychometric properties (Galli & Gonzalez, 2015). This scale is comprised of 10 items, that are answered on a scale from 0 (not true at all) to 4 (true nearly all the time). The items are grouped into a single factor representing the level of the respondent’s resilience. Several studies (e.g., Nartova-Bochaver et al., 2021) have supported validity and reliability of this measure in different countries.

#### Performance

The International Trail Running Association (ITRA) Performance Index is a tool for ranking athletes based on their sport performance level, and it has been used to compare the speed of trail runners around the world (Méndez-Alonso et al., 2021). The scale has a maximum of 1000 points corresponding to the best possible performance (world record performance for that distance), considering the athlete’s finish time and specific race characteristics, namely distance, elevation gain/loss and average altitude (ITRA, 2022). This scale utilizes an indirect normative comparison method (based on the statistical analysis of a database of more than 5.3 million individual results), with the technical characteristics of the terrain also considered (ITRA, 2022). A general performance index is calculated from the weighted mean of the five best race results over the previous 36 months (permitting reliable statistical calculations and giving the possibility of an injured athlete continuing to appear), regardless of the distance of each race (ITRA, 2022). By finishing a certified ITRA race (from a minimum of 2 km to more than 190 km), the result will appear in the ITRA Performance Index (ITRA, 2022). The number of races finished by participants in this study varied between 1 to 69 (*M* = 15.94; *SD* = 13.01). In addition, the average distance (in kilometers) made in certified trail races was 971.12 and the average distance of positive ascents was 46.45 km, while the average distance of negative ascents was 45.90 km.

### Statistical Analysis

Descriptive statistics included means (and standard deviations) and bivariate correlations for studied variables. In addition, a two-step maximum likelihood approach (ML) (Kline, 2016) in AMOS 27.0 was performed. A confirmatory factor analysis (CFA) first tested the psychometric properties of the measurement model, including its convergent and discriminant validity and composite reliability (Hair et al., 2019). Convergent validity was assessed via average variance extracted (AVE), considering values higher than or equal .50 as adequate (Fornell & Larcker, 1981). Discriminant validity was estimated through the square correlations between factors, and it was considered adjusted when the square correlations were below the AVE of each factor (Hair et al., 2019). Additionally, the internal consistency of each of the latent variables under study was calculated, from the composite reliability (Raykov, 1997), assuming as a cut-off value for adequacy coefficients, ≥.70 (Hair et al., 2019; Raykov, 1997). Next, a structural model was established to test the hypothesis. The model´s fit for both the measurement model and the structural model was observed through the traditional goodness-of-fit indexes. Specifically, we used the Comparative Fit Index (CFI) and Tucker-Lewis Index (TLI) and the absolutes of the Standardized Root Mean Residual (SRMR) and Root Mean Square Error of Approximation (RMSEA) with a confidence interval (CI 90%), as recommended by several authors (Kline, 2016; Hair et al., 2019; Byrne, 2016; Marsh et al., 2004) and with the following adopted cut-off values: CFI and TLI ≥ .90; RMSEA and SRMR ≤ .08 (Kline, 2016; Hair et al., 2019; Byrne, 2016; Marsh et al., 2004). Standardized direct and indirect effects on the dependent variable were also analyzed. The significance of direct and indirect effects was analyzed using a bootstrap resampling procedure (1000 bootstrap samples), through a 95% CI. The indirect effect was considered significant (≤0.05) if the 95% CI did not include zero (Williams & Mackinnon, 2008). We chose to consider confidence intervals rather than the probability of significance (*p*-value) due to recent evidence of mediation without a significant relationship between variables (Hayes, 2018).

## Results

### Preliminary Analysis

The Full Information Robust Maximum Likelihood (FIML) was used to handle the small amount of missing data at the item level (missing at random = 4%) as proposed by Enders (2010). Additionally, no outliers (univariate and multivariate) were identified. Item-level descriptive statistics indicated no deviations from univariate normality because skewness and kurtosis assumptions of the data distribution were comprised between −2 and +2 and −7 and +7, respectively (Hair et al., 2019). Mardia’s coefficient for multivariate kurtosis (47.83) exceeded expected values (5.0) for the assumption of multivariate normality (Byrne, 2016).

Therefore, the Bollen-Stine bootstrap on 2000 samples was employed for subsequent analysis (Nevitt & Hancock, 2001). Finally, the collinearity diagnosis was checked using variance inflation factor (VIF) and tolerance tests and these results revealed values between 1.56 to 1.88 for VIF and 0.38 to 0.77 for the tolerance test, demonstrating acceptable conditions for regression analysis (Kline, 2016; Hair et al, 2019). Then, descriptive statistics and bivariate correlations were calculated for all variables under analysis.

Descriptive statistics showed that the participants presented scores above the midpoint for the measures of MT and resilience. Looking at bivariate correlations, positive and significant associations were found between all variables under analysis, specifically including these observations: (a) MT was positively associated with both resilience and performance; and (b) resilience was positively associated with performance. The measurement model including the factors MT, resilience, and performance variables, exhibited an adequate fit to the data: χ^2^ = 150.01 (74); *BS-p* = .003; CFI = .953; TLI = .942; RMSEA = .058 90% (.045, .071) and SRMR = .042, since CFI and TLI were above and SRMR and RMSEA were below the previous reported cut-off values.

As seen by the CR coefficients, each factor showed scores above the cutoff (>.70), revealing adequate internal consistency. Based on the results of the measurement model and reliability analysis, convergent and discriminant validity were calculated. Convergent validity was achieved, since the AVE scores were above the acceptable cut-off values, as seen in Table 1. According to the squared correlations and AVE scores, all factors demonstrated adequate discriminant validity since the squared correlations of each latent variable were lower than the AVE scores in each latent variable. The results provide preliminary support to conduct SEM analysis and examine the direct between mental toughness and resilience with performance and indirect effect between mental toughness and performance via resilience.

**Table 1.** Descriptive Statistics, Bivariate Correlation, Convergent and Discriminant Validity, and Composite Reliability of the Participants Responses to MT and Resilience Measures and Their Trail Running Performance.

	*M*	*SD*	1	2	3	AVE	CR
1. MT	3.09	.48	1		—	.67	.82
2. Resilience	3.12	.54	.62**	1	—	.56	.84
3. Performance	650.91	543.87	.23**	.13**	1	—	—

*Note*. MT = mental toughness; *M* = mean; *SD* = standard deviation; AVE = average variance extracted; CR = composite reliability.

**Figure 1.**
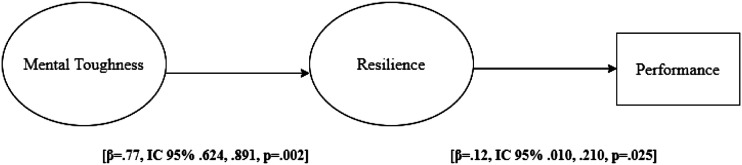
Standardized Direct Effects.

The results from the SEM analysis showed that the structural model provided an acceptable fit to the data, with *χ*^*2*^ = 150.01 (75); *BS-p* = .003; CFI = .954; TLI = .944; RMSEA = .057 90% (.044, .070) and SRMR = .042 since CFI and TLI were above and SRMR and RMSEA were below the previous reported cut-off values. Standardized direct effects revealed positive associations (see Figure 1) between variables. Specifically, MT displayed a significant association with resilience; and resilience was significantly associated with performance.

The indirect regression paths showed that MT was positively associated with performance, with resilience a possible mediator (*β* = .09 IC = .010, .168; *p* = .02) in this relationship. In total, considering direct and indirect effects the model explained 21% of the performance variance in trail runners.

## Discussion

In this study we aimed to analyze the associations across mental toughness, resilience, and athletic performance in Portuguese trail runners. Overall, our hypothesis was confirmed and will be discussed below in the context of existing literature.

The positive association found between MT and resilience was in line with previous research in which Nicholls et al. (2015) found significant associations between MT and resilience, concluding that “mentally tough athletes are able to excel under pressure.” MT, in the presence of other psychological constructs, can distinguish performance (not just coping) under stressful circumstances. Similarly, Gucciardi et al. (2008) found, through qualitative investigation, that “mentally tough athletes are resilient,” and Cowden et al. (2016) found a strong positive association in competitive South African Tennis players between MT and resilience, further affirming the conceptual similarities between the two constructs. Our standardized direct effects analysis also revealed a positive association between resilience and performance, in line with Hosseini and Besharat’s (2010) finding that resilience predicted athletes’ sporting achievement, psychological well-being and distress.

Our indirect regression analysis revealed a positive association between MT and athletic performance, with resilience considered as a possible mediator. This result empathizes the close relationship between these two constructs and gives new input to the study of psychological constructs in athletic performance. Despite the close relationship between resilience and MT, resilience retains its uniqueness, including the conditions of positive adaptation and adversity that Galli and Gonzalez (2015) described. The decisive role of resilience in facing severe adversities that can occur outside of the sport context (Cowden et al., 2016) seems to give resilience a mediation role for “the enactment and maintenance of goal-directed pursuits” as Zeiger and Zeiger (2018) suggested. Notwithstanding limited knowledge of the conceptual association between MT and athletic performance, due to scarce other empirical results to date, our results are in line with reviews by Cowden et al., (2017) and Guszkowska and Wojcik (2021) who reported a positive correlation between MT and sporting performance across different sports, regardless of the participants’ age, gender, or skill levels. This result emphasizes that MT is a multifaceted construct that is a central prerequisite to excellent sport performance (Cowden et al., 2017).

Our results are also in line with Méndez-Alonso et al. (2021) who found that MT and resilience are psychological predictors of success in ultra-trail runners (i.e., there were better classification and race times in athletes with higher values of MT and resilience). Méndez-Alonso et al. (2021) also highlighted that ultra-trail showed higher values of MT and resilience than either athletes in other sports or sedentary individuals, and this was also previously reported by Galli and Gonzalez (2015). Furthermore, past studies found higher levels of psychological constructs among endurance athletes, especially MT (Aryanto & Larasati, 2020) and resilience (Roebuck et al., 2020), raising the question of whether these attributes are intrinsic characteristics or consequences of training and/or competing. Méndez-Alonso et al. (2021) stated that each race works as a means of training these psychological factors, increasing an athlete’s MT and resilience. This training aspect is probably due to the unpredictable conditions and specific characteristics of each trail running race. This is a characteristic that sports professionals should consider in assessments of MT and resilience at pre-testing, during a race, and post- race. Although, Brace et al. (2020) demonstrated high levels of MT in a sample of elite level ultra-marathon runners, they did not find performance effects during a race among those with higher MT values, possibly suggesting that other factors can influence performance more. These results reinforce the important role of possible indirect associations between psychological constructs in performance variance. Endurance sports demand mental and physical integration owing to the impact of fatigue prompted by the high effort sport performance requires (Diotaiuti et al., 2021). Specifically, in trail running, it is necessary to make a permanent adjustment to different conditions (e.g., elevation and climate make it imperative that the athlete control various pace, nutrition, posture, loneliness, and fatigue). However, these athletes present a more effective way of contending with unpleasant situations and perform more effectively, acting with self-awareness of their own effort and fatigue (Guszkowska & Wojcik, 2021). This fact makes trail running different from others sports or even from other kinds of running races, adding importance to the use of permanent psychological control adaptations to reach sports goals, highlighting the general importance of mental characteristics and the primordial role of psychological preparation (MT and resilience training) in sports (Guszkowska & Wojcik, 2021). Performance in these races is multifactorial, with many factors involved (Diotaiuti et al., 2021), and our results show the importance of just these two constructs, while other psychological social, and physiological factors may explain remaining variance in athletic performance. Notwithstanding these other unknowns, the complexity of trail running makes it an ideal target for future sports research, and for endurance sports.

Our findings show that MT, mediated by resilience, explained 21% of the total performance variance in trail runners, providing a new perspective of the possible importance to intervention training in psychological constructs for these kind of endurance efforts. Sports professionals should be aware that mental training should be an integrant part of a holistic psychosocial program (Fletcher & Sarkar, 2016).

### Limitations and Directions for Future Research

Although previous studies analyzed the association between MT, resilience, and performance, this is the first study to analyze simultaneously MT, resilience, and performance in trail runners. While the present study contributed new insights into these associated psychological constructs, some limitations should be addressed. First, we used a cross-sectional design that precludes us from determining causal relationships between these variables. Experimental studies are needed to examine the effects of mental toughness and resilience on athletic performance. Second, our data were limited to a Portuguese trail running sample and may not generalize well to athletes from other cultures and/or sport contexts. Third we collected these data only in one moment, namely at the middle of the competitive calendar, and there may be variations in these results if data were collected at other times.

Future investigators should analyze MT and resilience during a competitive race calendar, taking into consideration the possible changes that may occur during a season. Additionally, the association between these and other constructs normally related, should be studied directly and indirectly, using not only cross-sectional but experimental designs. To obtain greater generalizability, future studies might be applied toward participants in other sports and cultures. Finally, investigations of whether or how these psychological constructs might be trained and enhanced should be part of future research.

## Conclusion

There is a growing appreciation for the importance that psychological preparation should assume in endurance sports and, specifically, in trail running, characterized by unpredictable and stressful conditions that makes variance in performance excellence multifactorial. These athletes must make permanent adjustments to different conditions higher performance has been associated with higher values of such psychological constructs as MT and resilience. Our findings showed that these constructs explained 21% of trail runners’ performance variance when considering their direct and indirect effects. This, highlights the close relationship between these two constructs and their joint influence on performance variation, contributing to a holistic view of athletic performance. Psychological training in endurance sports practice, especially including MT and resilience training, would seem advantageous obtaining better performances. These training programs should consider the cultural and contextual attributes of each sport and social and the athletes’ environmental context.
